# Development of gut microbiota composition in captive Asian elephants: a year-long analysis

**DOI:** 10.1038/s41598-026-46586-8

**Published:** 2026-04-01

**Authors:** Sarisa Klinhom, Chanon Kunasol, Sirawit Sriwichaiin, Sasiwan Kerdphoo, Nipon Chattipakorn, Siriporn C. Chattipakorn, Chatchote Thitaram

**Affiliations:** 1 Center of Elephant and Wildlife Health Animal Hospital, Faculty of Veterinary Medicine, Chiang Mai University, Chiang Mai, 50100 Thailand; 2 Center of Excellence in Cardiac Electrophysiology Research, Chiang Mai University, Chiang Mai, 50200 Thailand; 3 Neurophysiology Unit, Cardiac Electrophysiology Research and Training Center, Faculty of Medicine, Chiang Mai University, Chiang Mai, 50200 Thailand; 4 Cardiac Electrophysiology Unit, Department of Physiology, Faculty of Medicine, Chiang Mai University, Chiang Mai, 50200 Thailand; 5 The Academy of Science, The Royal Society of Thailand, Bangkok, Thailand; 6 Department of Oral Biology and Diagnostic Sciences, Faculty of Dentistry, Chiang Mai University, Chiang Mai, 50200 Thailand; 7 Faculty of Veterinary Medicine, Chiang Mai University, Chiang Mai, 50100 Thailand; 8 Center of Elephant, Wildlife, and Companion Animals Research, Chiang Mai University, Chiang Mai, 50100 Thailand; 9 Department of Companion Animals and Wildlife Clinics, Faculty of Veterinary Medicine, Chiang Mai University, Chiang Mai, 50100 Thailand

**Keywords:** Gut microbiome, Elephant calf, Elephant’s health, Elephant’s diets, Microbiology, Zoology

## Abstract

The establishment of gut microbiota is vital for the health and development of elephant calves, especially during their first year of life. This study investigates the transformation of the gut microbiome in captive Asian elephant calves, focusing the influence of maternal milk and subsequent dietary transitions on microbial colonization. A single meconium was collected at birth, followed by monthly fresh fecal samples during the first year. Mothers’ fecal samples were also collected when calves reached three months of age. Gut microbiota profiling was conducted using 16 S rRNA gene sequencing. Meconium exhibited a diverse microbiota with moderate richness at birth. Richness subsequently declined in the monthly fecal samples, reaching its lowest point by three months of age. Firmicutes, Actinobacteriota, and Bacteroidota were the dominant bacterial phyla throughout the first year. A major shift was observed around 7 to 8 months of age with emergence of the archaeal genus *Methanobrevibacter* from phylum Euryarchaeota, which subsequently declined by 11 to 12 months. By this age, the calf microbial community had become more similar to that of adult elephants; however, species richness and diversity remained lower, indicating that microbial maturation was still ongoing. These findings advance our understanding of gut microbiota development in elephant calves and underscore the critical role of dietary management in promoting gut health and survival, providing essential data for captive conservation and management strategies.

**Supplementary Information:**

The online version contains supplementary material available at 10.1038/s41598-026-46586-8.

## Introduction

The health status of Asian elephant calves (*Elephas maximus*) is a critical concern due to the high juvenile mortality rate, which significantly compromises the sustainability of captive populations. Despite the provision of optimal nutrition and veterinary care in captivity, pre-weaning mortality rates remain alarmingly elevated, with reported figures of approximately 30% in North America^1^ and 25.6% in Myanmar^2^. Prior research has identified multiple factors contributing to calf mortality, including physical injuries, accidents, maternal agalactia, malnutrition, neonatal weakness, and infections such as elephant endotheliotropic herpesvirus (EEHV)^2,3^. The early postnatal period is particularly crucial for the development of health parameters, notably immune system maturation and gut microbiota colonization^4^, which are essential for the successful transition of calves into healthy weanlings and ultimately to enhance survival rates.

As herbivores with hindgut fermentation, elephants’ diets mainly consist of grasses, fruits, and leaves. Elephant health is heavily dependent on gut microbiota, which aids in efficiently digesting plant material and extracting energy, including short-chain fatty acids (SCFA) and amino acids^5^, making their gut microbiomes diverse and complex compared to other mammals^6^. In humans^4,7–9^ and in elephants^10^, the gut microbiota in infants are notably more dynamic and less diverse than in adults, exhibiting a decreased number of bacterial species. The development of the infant gut microbiome is a highly complex process, susceptible to changes in diet, environment, and other factors, e.g. antibiotic use^11,12^. During infancy, maternal milk significantly impacts the colonization of infant microbiomes both directly, by providing beneficial microbes through mechanisms of maternal microbial transmission, and indirectly, through various bioactive factors that shape microbial growth and activity, ultimately contributing to the infant’s health and development^13^. Maternal milk contains a range of bioactive components, such as milk oligosaccharides, lactose, and various fatty acids, which promote the growth of beneficial microbes while inhibiting pathogenic bacteria^14^. These oligosaccharides, in particular, act as prebiotics, selectively nourishing beneficial gut bacteria and fostering their proliferation^15^. Maternal milk contains immunological factors, including secretory immunoglobulin A (IgA) and antimicrobial proteins, which further protect the infant against infections and support the development of a balanced microbiome^16^. Although these functions have been extensively described in human milk, elephant milk exhibits comparable properties. Both African and Asian elephant milk contain exceptionally high concentrations of free oligosaccharides, including fucosylated and sialylated structures also found in human milk^17,18^. These components might exert probiotic, anti-infective, and immunomodulatory functions in elephant calves.

In Asian elephants, the weaning process typically begins around 12 to 14 months of age, with calves gradually shifting from milk consumption to solid foods such as grass and leaves^19–22^. This transition from maternal milk to solid food can take several years. Coprophagy, the consumption of feces from mothers or herd members, begins around 4 to 6 months and helps calves acquire microbes essential for digesting plant material^23^. These behaviors support the gradual establishment of an adult-like gut microbiota even before complete weaning. Captive calves are usually fully weaned by about three years of age, whereas wild calves are reported to be completely weaned at approximately four years of age^2^. Earlier weaning in captivity may negatively influence the establishment of a stable gut microbiota and the development of immune functions essential for long-term health and survival, potentially contributing to the higher mortality often observed in captive populations.

Understanding how the gut microbiota transforms during infancy is crucial for enhancing the survival prospects of young calves, particularly in the face of challenges such as undernutrition, infectious diseases, and antibiotic treatments, all of which can directly impact gut microbiota communities. Recent research has highlighted the developmental trajectory of the infant gut microbiota as a significant area of interest across various species, including elephants. Notably, a study on infant Asian elephants revealed that during the first five weeks of life, the gut microbiota is predominantly composed of *Lactobacillales*, which have been positively correlated with milk components such as lactose, sugars, fatty acids, and threonine^22^. Despite the critical role of a healthy gut microbiome in the overall health and development of elephants, the specific factors influencing its growth during infancy and early life remain incompletely understood. This study aims to investigate the alterations in the gut microbiota of elephant calves throughout their first year of life. Using Next-Generation Sequencing (NGS) technology to sequence the 16 S ribosomal RNA (rRNA) gene, we seek to characterize the microbiota community’s transition in pre-weaning elephants. We hypothesize that during the first year of life, although elephant calves are not yet fully weaned, their gut microbiota gradually shifts in response to the introduction of solid plant-based foods and coprophagy, including an increase in fiber-degrading bacteria. We expect these early dietary exposures to be key drivers of microbiome maturation. Understanding these microbial shifts is important for identifying factors that may influence calf health and support efforts to reduce calf morbidity and mortality.

## Results

The longitudinal study of elephant calves revealed dynamic changes in gut microbiota and their predicted functional implications for elephant health. The comprehensive sampling strategy enabled the evaluation of microbial communities across different age groups, as well as a comparison between neonatal and maternal microbiota.

### Diversity and age-related dynamics of the gut microbiome in elephant calves and their mother

**Fig. 1:**
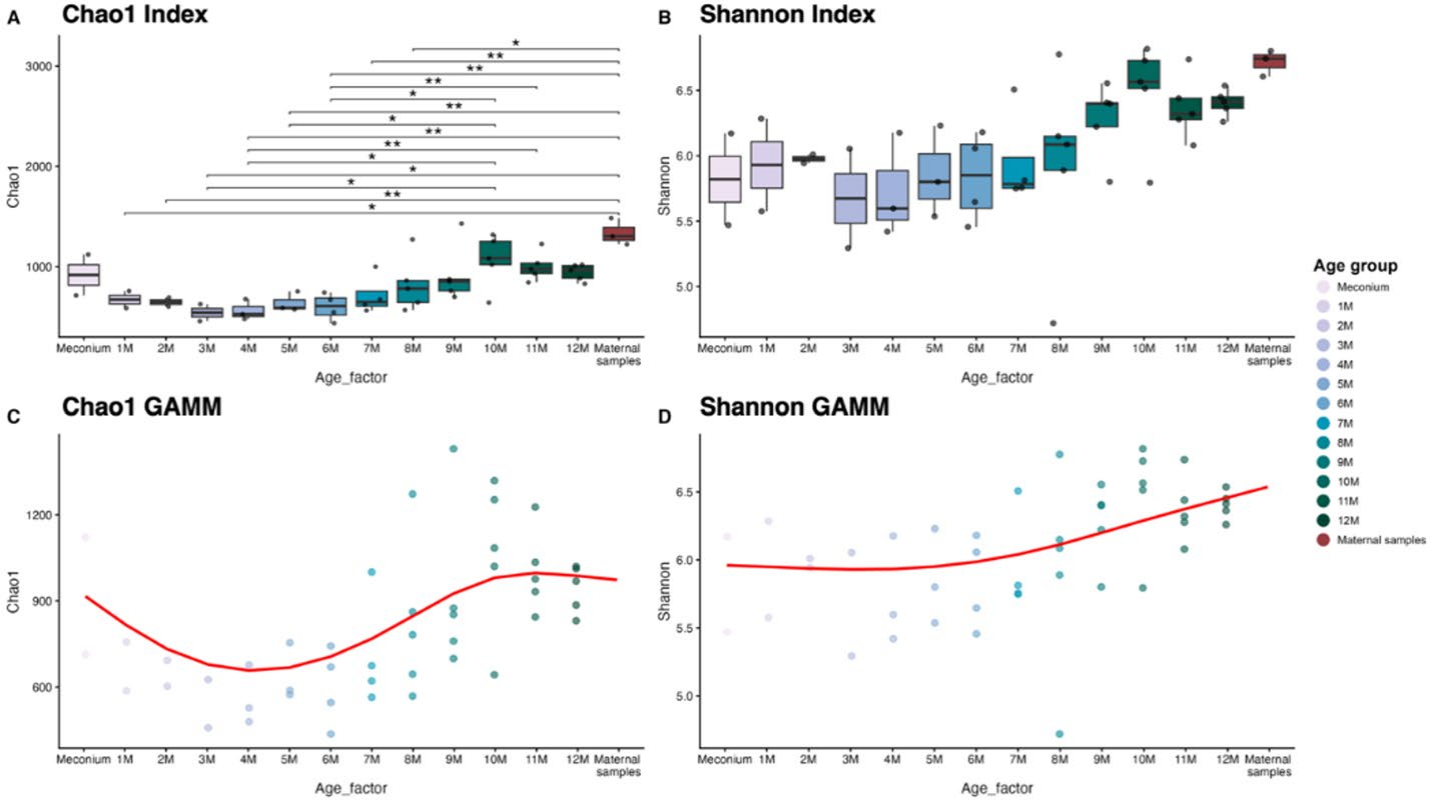
Alpha diversity of the gut microbiota in elephant calves across meconium samples, monthly intervals, and maternal samples. Diversity was assessed using **(A)** Chao1 richness and **(B)** Shannon diversity indices. Statistical differences were evaluated using a Linear Mixed-Effects Model (LMM), with * indicating *p* < 0.05 and ** indicating *p* < 0.01. Trends in microbial diversity over time are shown using Generalized Additive Mixed Models (GAMMs) for (C) Chao1 and (D) Shannon indices. Higher values indicate increased richness or evenness in the microbial community.

We compared the alpha diversity of the elephant calves’ gut microbiota at monthly intervals during the first year of life and their mothers using the Chao1 and Shannon indices Linear Mixed Models (LMM) with pairwise comparisons for each index and Generalized Additive Mixed Models (GAMMs) were applied to evaluate age-related changes in alpha diversity while accounting for repeated measures within individuals (Fig. 1). The Chao1 index, representing microbiota richness based on amplicon sequence variants (ASVs), increased with age and differed significantly among age groups (LMM, F_13, 29.1_ = 3.70, *p* = 0.0016) (Fig. 1A, Supplementary Table 1). Pairwise comparisons showed no significant differences between meconium and older ages or the mother. Significant differences in alpha diversity were observed between age intervals, with richness at 3 to 6 months being consistently significantly lower than at 10 to 11 months (3 m vs. 10 m, *p* = 0.028; 4 m vs. 10 m, *p* = 0.018; 4 m vs. 11 m, *p* = 0.029; 5 m vs. 10 m, *p* = 0.039; 6 m vs. 10 m, *p* = 0.015; 6 m vs. 11 m, *p* = 0.028). Since no significant differences were detected among ages within either the 3 to 6-month or 10 to 11-month intervals, this supports internal homogeneity within each interval. These findings suggest that mid-first year of life (3 to 6 months) exhibited lower richness, followed by a marked increase as calves approached one year of age (10 to 11 months). In the Chao1 index, maternal microbiota exhibited significantly higher richness than that of infants aged 1–8 months (*p* < 0.05), suggesting that the gut microbiome during this period was undergoing gradual maturation toward the adult profile.

Shannon diversity gradually increased with age. Calves aged 10–12 months showed higher diversity than younger calves (meconium and 1–9 months), approaching the levels observed in their mothers (Fig. 1B). Mothers consistently exhibited the highest richness and diversity. However, after multiple-testing correction, no statistically significant differences were detected among age groups, including between 10–12-month-old calves and maternal samples (Supplementary Table 2). GAMMs analyses revealed that species richness (Chao1, Fig. 1C) exhibited more dynamic changes than overall evenness (Shannon, Fig. 1D) during early gut microbiota development, with both metrics stabilizing toward maternal-like levels by 10 to12 months. GAMMs of Chao1 richness decreased from ~ 916 at birth to ~ 660 by 3 to 4 months, then increased to ~ 988 by 12 months, showing a nonlinear trajectory (edf = 3.54) consistent with early microbial filtering followed by diversification (Supplementary Table 3). GAMMs of Shannon diversity increased gradually and slightly nonlinearly with age (edf = 2.06), reflecting slow maturation of community evenness, with predicted values rising from ~ 5.96 at birth to ~ 6.46 by 12 months (Supplementary Table 3).

**Fig. 2:**
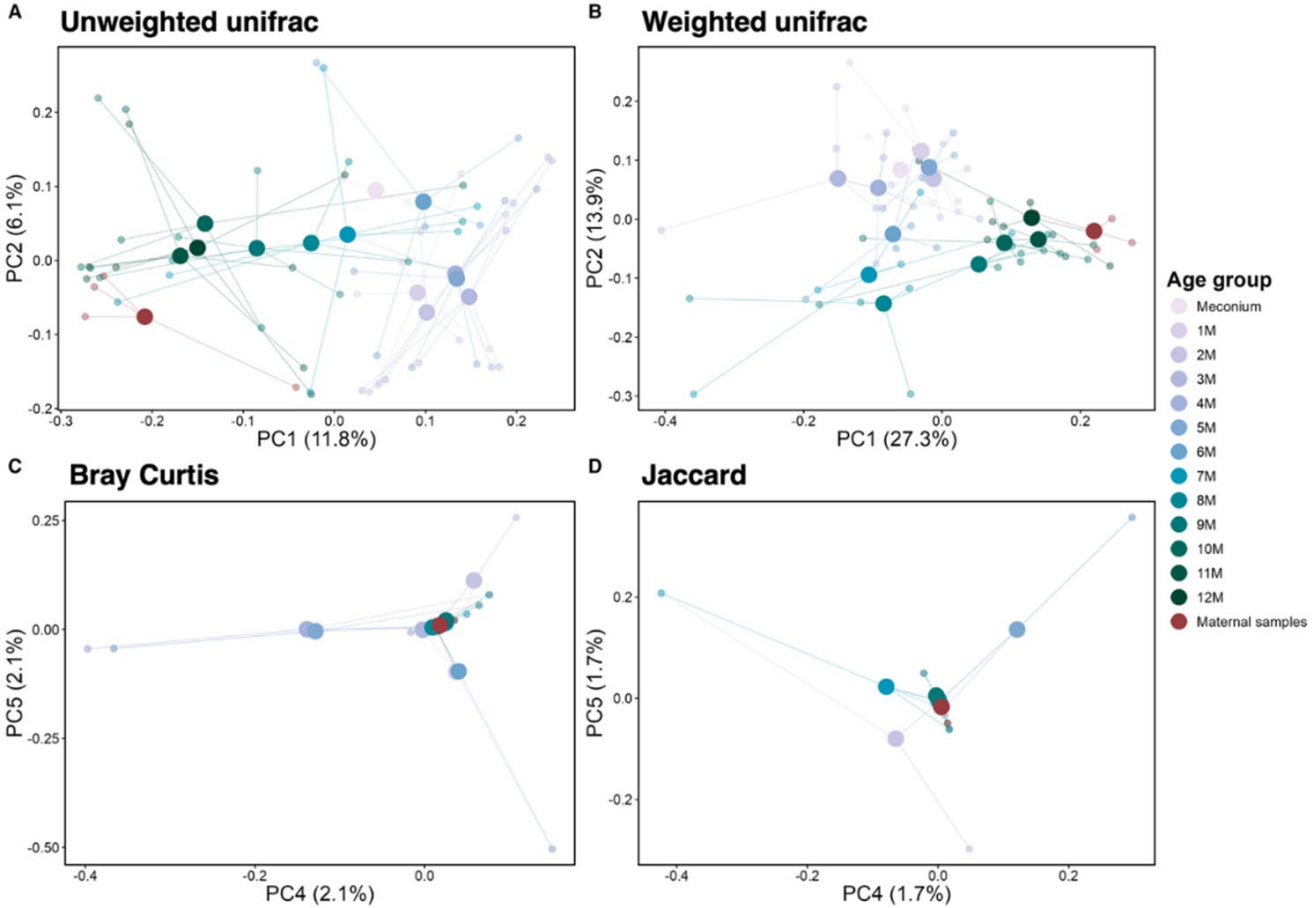
Principal Coordinate Analysis (PCoA) plots illustrating differences in gut microbiota composition across meconium samples, monthly intervals, and maternal samples. Beta diversity was assessed using **(A)** Unweighted UniFrac, **(B)** Weighted UniFrac, **(C)** Bray–Curtis, and **(D)** Jaccard distance metrics. Small points represent individual samples colored by age group. Large points indicate group centroids (mean PcoA coordinates for each age group), and connecting lines represent the distance of each sample from its group centroid (distance-to-centroid ordination spider plot). Full statistical comparisons for each distance metric are provided in Supplementary Tables 4 to 7.

Microbial community composition was analyzed across meconium samples, monthly interval fecal samples, and maternal samples using Unweighted UniFrac, Weighted UniFrac, Bray–Curtis, and Jaccard distances (Fig. 2). Significant shifts in microbiota composition were identified using LMM and pairwise comparisons for each distance metric. Unlike alpha diversity, which was grouped into biologically defined age intervals due to stable within-group variation, beta diversity showed continuous changes across months and was therefore analyzed at monthly interval. Unweighted UniFrac (Fig. 2A) and Weighted UniFrac (Fig. 2B) distances revealed significant changes, capturing phylogenetic differences in community composition based on presence/absence and relative abundance, respectively. Unweighted UniFrac PC1 revealed rapid microbiota changes from birth to 6 months, with significant differences between meconium samples and those aged 2 to 6 months (*p* < 0.05; Supplementary Table 4). After 6 to 7 months, microbiota stabilized and resembled older calves and maternal composition. Unweighted UniFrac PC2 showed no significant age-related changes, suggesting that this axis captures variability unrelated to age. For Weighted UniFrac PC1 maternal microbiota differed significantly from meconium samples (*p* = 0.038; Supplementary Table 5). Weighted UniFrac PC2 exhibited age-dependent changes in infant microbiota, with significant differences between meconium samples and those aged 6 to 8 months (meconium vs. 7 m, *p* = 0.024; meconium vs. 8 m, *p* = 0.004). Weighted UniFrac demonstrates that while maternal microbiota is distinct (PC1), the infant gut microbiota gradually shifts with age (PC2), likely reflecting dietary transitions and microbiota maturation.

Additionally, with Bray–Curtis distances (Fig. 2C), which assess quantitative dissimilarity, PC2 revealed that 3-month-old calves differed significantly from all other age groups and from mothers (*p* < 0.05; Supplementary Table 6), possibly reflecting changes in dietary or digestive system. In contrast, Jaccard distances (Fig. 2D), a qualitative measure, showed no significant differences across the first year or between calves and the mother (Supplementary Table 7).

### Maternal–calf associations and environmental effects on calf gut microbiota

**Fig. 3:**
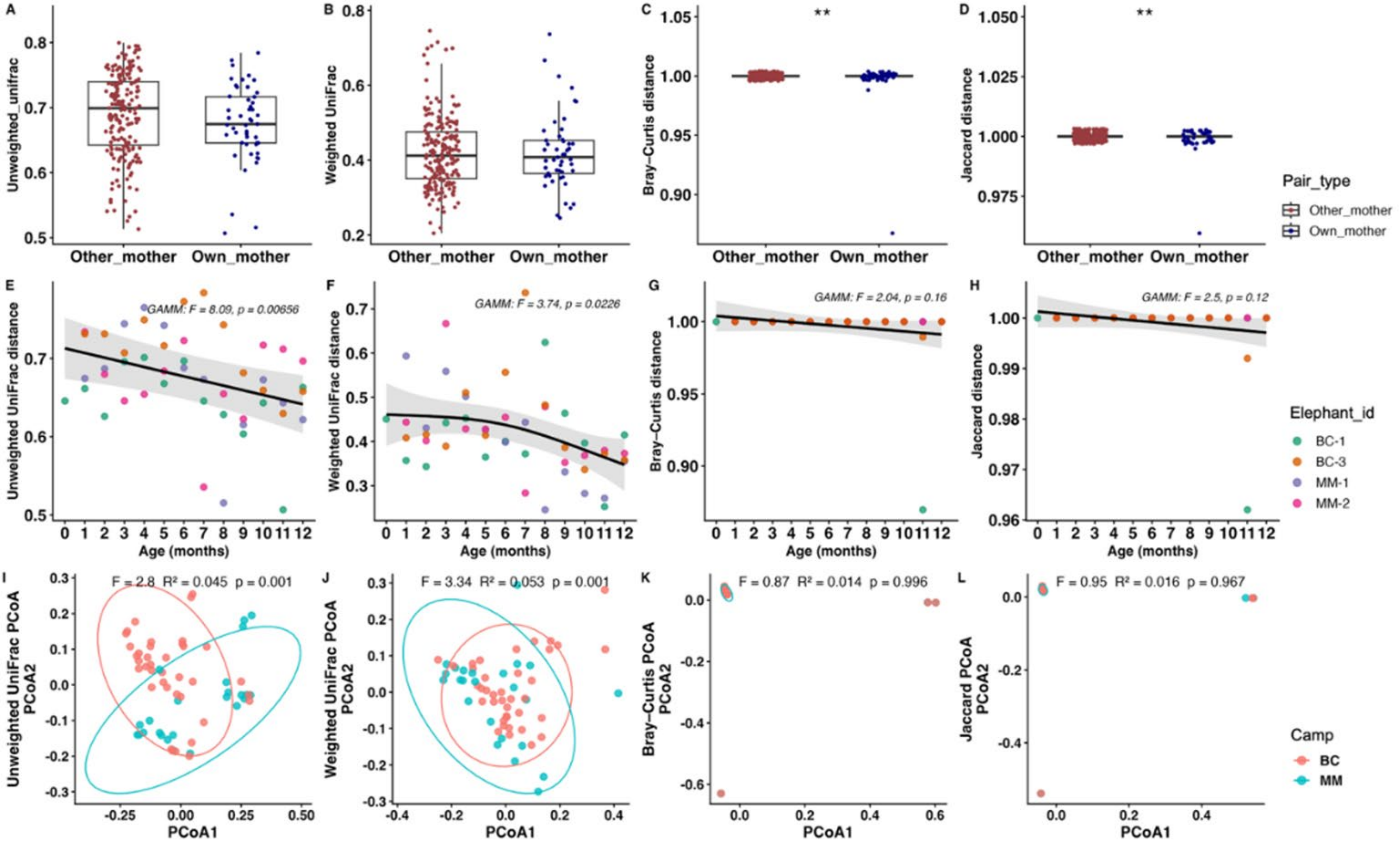
Mother–calf gut microbiota similarity and between-camp beta-diversity. **(A–D)** Boxplots show pairwise beta-diversity distances between calf samples and their biological mothers (own mother) compared with distances between calves and other adult females (other mothers), calculated using **(A)** Unweighted UniFrac, **(B)** Weighted UniFrac, **(C)** Bray–Curtis, and **(D)** Jaccard metrics. Using Wilcoxon rank-sum tests and linear mixed-effects models with calf identity included as a random effect (**p < 0.01). **(E–H)** Age-related trajectories of calf–mother beta-diversity distances across the first year of life based on **(E)** Unweighted UniFrac, **(F)** weighted UniFrac, **(G)** Bray–Curtis, and **(H)** Jaccard. Shaded areas indicate 95% confidence intervals. **(I–L)** PCoA of gut microbiota from elephants in two camps based on (I) Unweighted UniFrac, **(J)** Weighted UniFrac, **(K)** Bray–Curtis, and **(L)** Jaccard.

To evaluate whether any particular calf harbored a gut microbiota more similar to its own mother than to other adult females, pairwise beta-diversity distances between calves and mothers were compared using Bray–Curtis, Jaccard, unweighted UniFrac, and weighted UniFrac metrics (Fig. 3). Calf–own mother pairs exhibited significantly lower Bray–Curtis distances than calf–other adult female pairs (Wilcoxon *p* = 0.004; LMM *p* = 0.029; Fig. 3C). Similarly, Jaccard distances were significantly lower in calf–own mother pairs (Wilcoxon *p* = 0.004; LMM *p* = 0.017; Fig. 3D), indicating modest but consistent differences in community composition and the presence of shared taxa. In contrast, no significant differences were detected for Unweighted UniFrac (Wilcoxon *p* = 0.104; LMM *p* = 0.128; Fig. 3A) or Weighted UniFrac distances (Wilcoxon *p* = 0.966; LMM *p* = 0.706; Fig. 3B). Together, these results suggest a subtle maternal-associated signal in gut microbiota composition, while phylogenetic relatedness and abundance-weighted phylogenetic similarities were comparable across all adult females.

Age-related changes in calf gut microbiota similarity to their mothers were assessed using GAMMs applied to pairwise beta-diversity distances across the first year of life, with calf identity included as a random effect. Calf age was significantly associated with both Unweighted UniFrac (F = 8.09, *p* = 0.0066; Fig. 3E) and Weighted UniFrac distances (F = 3.74, *p* = 0.0226; Fig. 3F), whereas no significant age-related trends were detected for Bray–Curtis (F = 2.04, *p* = 0.16; Fig. 3G) or Jaccard distances (F = 2.50, *p* = 0.12; Fig. 3H). These findings indicate age-dependent phylogenetic restructuring of the calf gut microbiota relative to their mothers, while overall community composition and shared taxa remained relatively stable.

Moreover, differences in gut microbiota between the two elephant camps were evaluated using PERMANOVA based on multiple beta-diversity metrics. No significant differences were observed with Bray–Curtis (R² = 0.014, *p* = 0.997; Fig. 3K) or Jaccard distances (R² = 0.016, *p* = 0.964; Fig. 3L), indicating similar overall community composition and taxonomic membership between camps. In contrast, both Unweighted UniFrac (R² = 0.045, *p* = 0.001; Fig. 3I) and weighted UniFrac (R² = 0.053, *p* = 0.002; Fig. 3J) showed significant differences, suggesting phylogenetically distinct microbial communities. Beta-dispersion analysis indicated no significant difference for Unweighted UniFrac (*p* = 0.424), supporting a genuine shift in community structure, whereas dispersion differed for Weighted UniFrac (*p* = 0.034), suggesting a partial influence of within-camp variability. All pairwise distance data and GAMM statistical outputs are provided in Supplementary Table 8.

### Development of gut microbiota during the first year of life

**Fig. 4:**
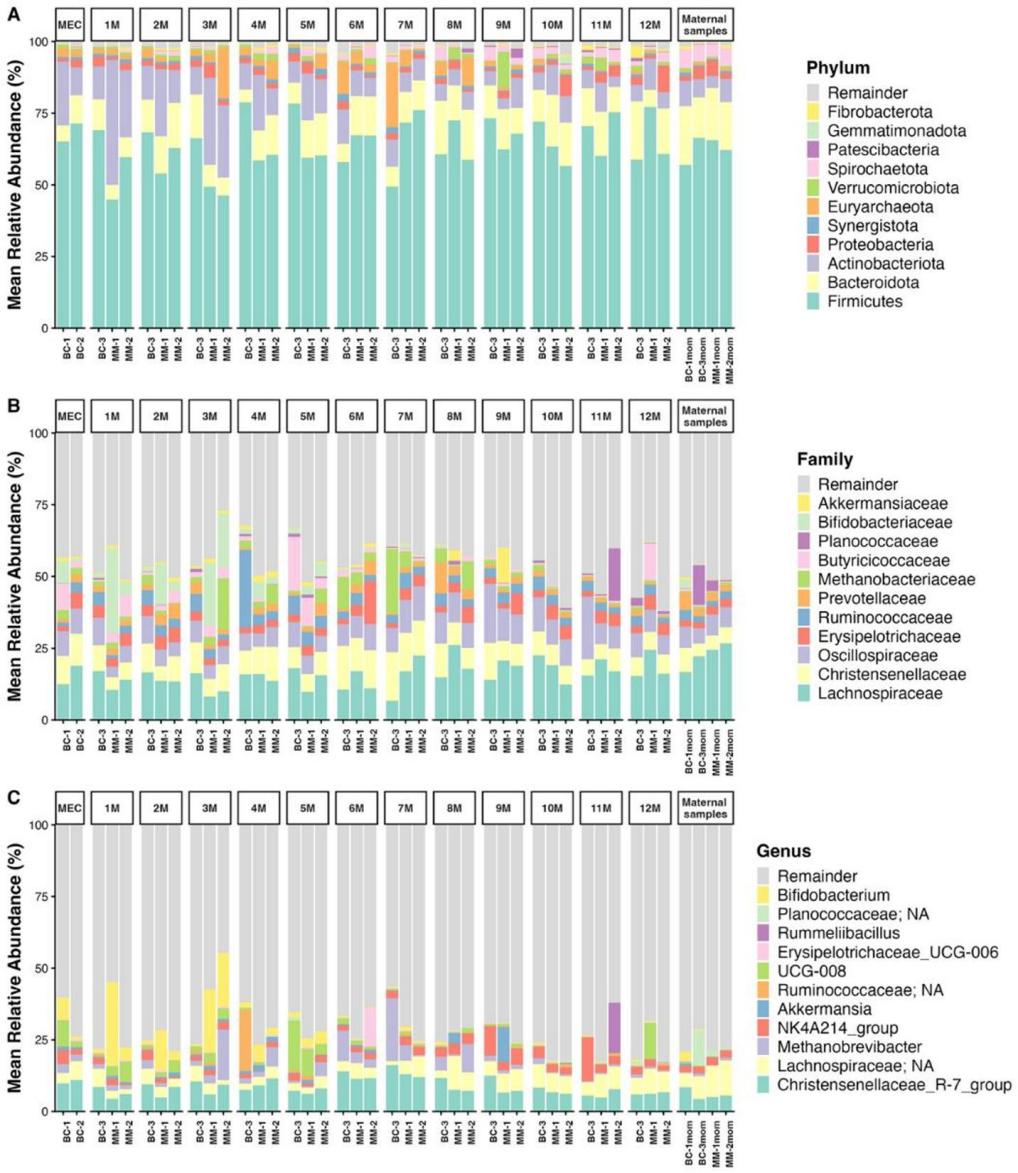
Relative abundance of the top 11 bacterial taxa in gut microbiota composition across meconium samples, monthly intervals, and maternal samples at the **(A)** phylum, **(B)** family, and **(C)** genus levels. Less abundant taxa were grouped as “Remainder.” The figure highlights major shifts in dominant taxa throughout the first year of life. “MEC” refers to meconium samples, and “M” refers to monthly age.

**Fig. 5:**
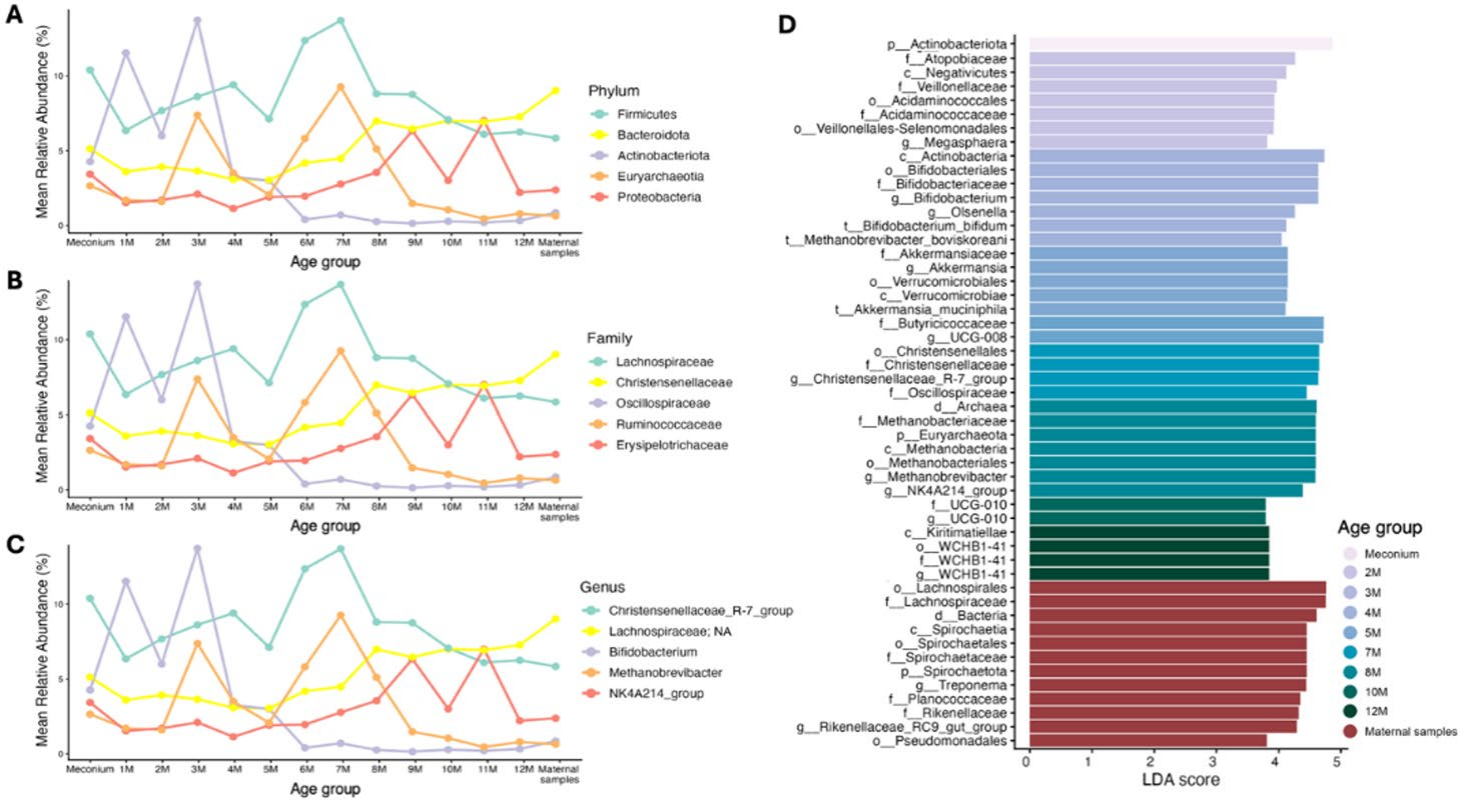
Temporal changes in the mean relative abundance of the top five most abundant taxa at the **(A)** phylum, **(B)** family, and **(C)** genus levels across meconium samples, monthly intervals, and maternal samples. **(D)** LEfSe analysis identifies indicator taxa enriched in specific age groups or maternal samples using an LDA score > 4. Taxonomic annotations follow the format: p, phylum; c, class; o, order; f, family; g, genus; t, species.

The gut microbiota of elephant calves showed considerable variation throughout the first year of life, with notable differences among individuals (Fig. 4). At the phylum level, Firmicutes dominated during the first four months, followed by Actinobacteriota (Figs. 3A and 4A). As the calves aged, Bacteroidota increased in abundance, becoming the second most dominant phylum, resembling the microbial composition of their mothers. Additionally, Proteobacteria levels increased with age, while Actinobacteriota and Euryarchaeota declined (Figs. 4A and 5A).

At the family and genus level, *Bifidobacterium* (family *Bifidobacteriaceae*) was predominant during the first three months but gradually decreased with age (Figs. 4B-C and 5B-C). From four to nine months, *Christensenellaceae*_R-7_group (family *Christensenellaceae*) became the most prevalent (Fig. 5B, C). *Methanobrevibacter* (family *Methanobacteriaceae*) increased in relative abundance between three and eight months, indicating a shift in microbial composition. As the calves matured, fiber-degrading genera became more prominent, particularly unclassified genera within *Lachnospiraceae* and NK4A214_group (family *Oscillospiraceae*) (Fig. 5B-C), which are involved in complex plant fiber degradation. These changes reflect the dietary transition from milk to fiber-rich solid foods.

In elephant meconium (M-0), the gut microbiota was predominantly composed of the phylum Firmicutes, followed by Actinobacteriota, Bacteroidota, and Euryarchaeota, resembling the microbial composition observed during the first four months of life (Figs. 4A and 5A**)**. At the genus level, however, the microbiota in meconium stool was markedly different from that of later monthly fecal samples and maternal samples, *Christensenellaceae*_R-7_*group* as the dominant genus, followed by unclassified genera within *Lachnospiraceae* and *Bifidobacterium* (Fig. 5C). Moreover, bacteria present in infant meconium were distinct from those in the maternal gut microbiome.

### Dynamic age-specific changes in the gut microbiota of elephants during the first year of life

LEfSe analysis revealed significant variations in the gut microbiota composition of elephants across different age groups during their first year of life. Fifty-one microbial taxa, identified using LDA scores > 4, indicated dynamic shifts in the gut microbial community over time (Fig. 5D).

At birth (0 months), the meconium of elephant calves was dominated by the phylum Actinobacteriota. By 2 months, there was a notable enrichment of the class Negativicutes, along with the order Veillonellales-Selenomonadales and Acidaminococcales, the families *Atopobiaceae*, *Veillonellaceae*, and *Acidaminococcaceae*. In 3-month-old elephants, the class Actinobacteria was dominant, particularly the order Bifidobacteriales, the family *Bifidobacteriaceae*, the genera *Bifidobacterium* and *Olsenella*, and the species *Bifidobacterium bifidum* and *Methanobrevibacter boviskoreani* were also prominent at this stage. By 4 months, the microbiota was enriched with the class Verrucomicrobiae, including the order Verrucomicrobiales, the family *Akkermansiaceae* and the genera *Akkermansia* and *Succiniclasticum*, with the specie *Akkermansia muciniphila* being particularly abundant. At 5 months, the family *Butyricicoccaceae* and the genus *UCG-008* were predominant. At 7 months, there was a significant increase in the order Christensenellales, along with the families *Christensenellaceae* and *Oscillospiraceae*, and the genus *Christensenellaceae_R-7_ group*. The gut microbiota of 8-month-old elephants was characterized by a dominance of the phylum Euryarchaeota, particularly the class Methanobacteria, the order Methanobateriales, the family *Methanobacteriaceae*, including the genus *Methanobrevibacter* and the *NK4A214 group*. At 10 months, the family and genus *UCG-010* were predominant. By 12 months, there was a significant increase in the class Kiritimatiellae and the order WCHB1-41. Additionally, the mothers of these elephant calves were enriched with plant degrading bacteria including with the phylum Spirochaetota, the class Spirochaetia, the order Spirochaetota, Lachnospirales, and Pseudomonadales, the families *Spirochaetaceae*, *Lachnospiraceae*, *Planococcaceae* and *Rikenellaceae*, including the genera *Treponema* and *Rikenellaceae_RC9_gut_group*. These findings highlight the dynamic and age-specific changes in the gut microbiota of elephants during the first year of life.

### Functional differences in gut microbiota during the first year of life in elephants

**Fig. 6:**
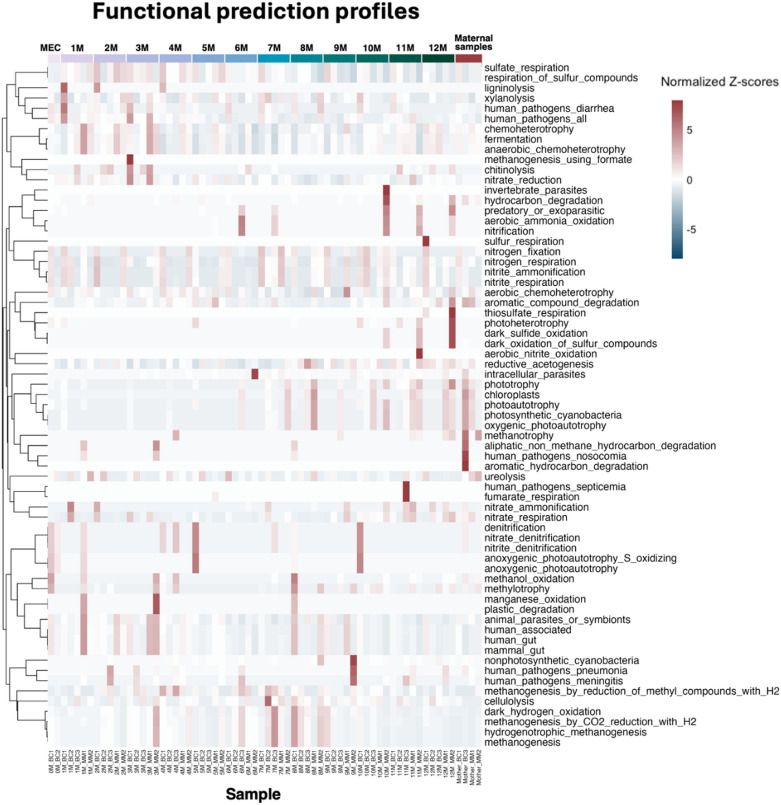
Heatmap of predicted bacterial functional profiles based on FAPROTAX analysis. The heatmap shows the distribution and relative abundance of functional categories across meconium samples, monthly age groups, and maternal samples, highlighting functional shifts associated with gut microbiota maturation during early development. “MEC” refers to meconium samples, and “M” refers to monthly age.

Understanding the functional maturation of the gut microbiota in elephant calves is crucial for insights into their early health development. These functional predictions were inferred from 16 S rRNA data using FAPROTAX rather than obtained through direct metagenomic sequencing. FAPROTAX functional prediction indicated potential shifts in microbial functions during the first year of life (Fig. 6). The predominant functions across all samples were fermentation and anaerobic chemoheterotrophy. Across meconium samples and fecal samples from infants aged 1 to 3 months, the gut microbiota was enriched in pathways characteristic of the early human gut microbiota, particularly those related to nitrate reduction. As the infants aged, a distinct shift was observed, with an increase in methanogenesis-related functions, particularly between 7 and 8 months of age. These functional transitions highlight the dynamic development of the gut microbiota and underscore the influence of early-life microbial changes on the health and growth of young elephants.

## Discussion

Upon birth, a complex microbial ecosystem begins to establish itself within the gastrointestinal tract of elephants. The development of the gut microbiota in captive Asian elephant calves over the first year of life followed a dynamic trajectory, highlighting key phases of microbial colonization and maturation. Our results demonstrated changes in both the diversity and functional capabilities of the gut microbiota as the calves matured, with their microbial communities increasingly resembling those of adult elephants, particularly their mothers.

In this study, two elephant meconium samples exhibited higher richness and evenness compared to those collected during the first four months of life and were composed of diverse bacterial taxa, suggesting very early microbial exposure and the initial establishment of the gut microbiota, potentially during late gestation or immediately after birth. However, because meconium samples were collected after birth and negative controls were not included, contamination from the birthing process or early environmental exposure cannot be excluded. Previous studies have suggested that fetal microbial exposure may begin before birth through maternal-derived microbial metabolites and possible microbial translocation via distinct pathways^24–26^. Commensal microbes or their components may cross the maternal intestinal barrier, enter the bloodstream, and reach the placenta or fetal gut, potentially priming the fetal immune system^25,26^. Therefore, this study cannot confirm that the microbiota detected in elephant meconium originated from in utero colonization. Nevertheless, further studies incorporating sterile sampling procedures and appropriate negative controls are required to investigate prenatal microbial colonization in elephants.

During the first six months of life, distance-based similarity analyses revealed a distinct separation between the microbial communities of neonatal elephants and those of older calves and adults. During this period, neonates harbored communities with lower species richness, consistent with previous studies showing that microbiota richness in elephant calves is initially low^10,22,27^. In this study, richness increased rapidly during the early postnatal period, reflecting the accumulation of microbial taxa, whereas the establishment of evenness and stability required more time. Shannon diversity tended to increase with age, but statistical analysis using LMM did not yield significant differences, likely due to small sample size and high variability across individuals, which may have obscured subtle changes. Beta diversity analyses further showed that microbial composition shifted rapidly during the first 6 to 8 months, with maternal microbiota distinct from newborns, while overall microbial membership remained relatively stable, suggesting that maturation involves changes in abundance rather than presence or absence. This pattern has been observed in other species, such as humans^28,29^ and horses^30^, where the early gut microbiota was highly dynamic and dominated by transient bacterial populations that diminish as the host ages.

Furthermore, this study demonstrated that elephant calves harbored gut microbial communities more similar to their own mothers than to other adult females, as indicated by significantly lower Bray–Curtis and Jaccard distances. In this study, during first year of life, mother–calf pairs were typically separated from the herd and maintained in enclosures to minimize tourist contact and external disturbance, allowing close maternal care. This management practice likely enhanced intensive mother–calf contact and facilitated early vertical microbial transmission, supporting a strong maternal influence on initial gut microbiota assembly through close physical contact, nursing, and shared microenvironments, consistent with maternal-associated microbial transmission reported in humans^31,32^. In contrast, the absence of significant differences in UniFrac-based metrics suggested that phylogenetic structure and abundance-weighted community patterns were not uniquely shaped by maternal association at this early stage. Although calves initially resembled their mothers in community composition, their gut microbiota gradually diverged and reorganized along phylogenetic lineages during first year of life. Together, these findings highlighted the combined roles of early maternal seeding and postnatal microbial succession in shaping the gut microbiota of captive Asian elephant calves. However, in some captive Asian elephant camps, calves may be reared by non-biological mothers (allomothers) or other herd members alongside their biological mothers, and in certain settings they are also frequently handled by mahouts or keepers^2,33^, which may alter early microbial exposure and influence gut microbiota development. Further studies across different management systems are warranted to evaluate their impacts on microbial transmission patterns and gut microbiota development in elephant calves.

At the taxonomic level, Firmicutes remained the dominant phylum in the gut microbiota of elephant calves throughout the first year of life. The early months were characterized by a high relative abundance of Actinobacteriota and a lower relative abundance of Bacteroidota. We observed a significant enrichment of milk-utilizing bacteria, specifically *Bifidobacterium* (family *Bifidobacteriaceae*) and *Akkermansia* (family *Akkermansiaceae*), in the gut microbiota of elephant calves. While our study did not directly analyze maternal milk components. Previous research in humans has shown that breast milk often contains viable *Bifidobacterium*, which may contribute to the initial establishment of the infant gut microbiota^34,35^. Both, *Bifidobacterium* and *Akkermansia* are well known for their ability to utilize milk oligosaccharides^36–40^, which were indigestible by the host. Notably, in Asian elephant milk, approximately 40% of the carbohydrate content was composed of milk oligosaccharides, a concentration considerably higher than that found in most other mammals^18^. This high oligosaccharide content likely played a pivotal role in shaping the early gut microbiota, especially during the first four months of life, when maternal milk constituted the primary diet of elephant calves. Moreover, research on elephant gut microbiota has suggested a dynamic interplay between breast milk components, such as lactose, low-abundance sugars (e.g., rhamnose), fatty acids, and threonine, and the early colonization of certain microbial groups such as *Lactobacillales*, which are prominent during the first few days of life^22^. This highlighted the importance of maternal milk in the development and maturation of the infant gut microbiota during the early stages of life.

The presence of nitrate-reducing bacteria in the gut microbiota of three-month-old elephant calves might be influenced by the high concentrations of nitrate and nitrite found in maternal milk. Human breast milk contains high concentrations of nitrate and nitrite in the early postpartum period, and these concentration might decrease as infant transitions to mature milk^41^, suggesting a dynamic relationship that might facilitate the establishment of beneficial gut microbiota. In elephant, it is suspected that milk provides essential substrates that support the colonization of specific gut bacteria capable of nitrate reduction. The ability of these bacteria to convert nitrate to nitrite could potentially support the gastrointestinal tract, potentially aiding in the development of a robust and diverse microbiome in the early life stage^42^. Additionally, the presence of nitrate-reducing bacteria might enhance the overall resilience of the gut ecosystem during a crucial developmental phase^43^, supporting nutrient absorption and immune function in the growing elephant calf. As breast milk composition changes over time, these shifts likely correlate with the evolving gut microbiota and the metabolic demands of the growing elephant.

In our study, we collected maternal fecal samples when the elephant calves were 2 months old to investigate the bacterial taxa associated with coprophagy in the calves. In elephant, coprophagy behaviour was typically observed in the first 4 to 6 months of life^23^. Coprophagy may contribute to shaping the gut microbiota of elephant calves, similar to observations in other herbivorous mammals^44,45^. The act of coprophagy facilitates the vertical transfer of microbiota from mothers to their young, supplying essential microbes that help stabilize the gut microbiome and aid in the uptake of essential amino acids, vitamins B and K, SCFA, and trace elements that are not fully absorbed^46^. However, our analysis did not identify specific bacterial taxa influencing this colonization process. This highlights a gap in understanding the microbial dynamics involved in gut maturation. Future studies should characterize the bacterial taxa in maternal feces and their effects on the infant gut microbiota, particularly during the early months when coprophagy is most prevalent. Although our study included fecal samples from both calves and their mothers, the dataset does not allow us to determine microbial transmission pathways or evaluate how maternal microbiota influence calf gut development. In addition, sampling was limited to the first 12 months of life, before the onset of weaning, preventing assessment of microbiome maturation during the weaning transition. Future longitudinal studies spanning the full weaning period and designed to track maternal–calf microbial transfer are needed to clarify how gut microbiota support successful weaning in captive populations.

Functional predictions revealed a transition in microbial activities during the first year of life, shifting toward more complex functions such as methanogenesis. This change was particularly evident in calves aged 7 to 8 months. The increased abundance of methanogenic bacteria at this stage suggests an important metabolic shift within the gut microbiota, likely reflecting the calves’ adaptation to a more fibrous diet. Methanogenesis, a process facilitated by archaea such as *Methanobrevibacter*, plays a key role in removing hydrogen (H₂) from the gut by converting it into methane (CH₄)^47,48^. This process was typically beneficial in preventing the accumulation of H₂, which could inhibit crucial metabolic reactions involved in fermentation^48^. The presence of methanogenic bacteria such as *Methanobrevibacter* suggested that the gut microbiota was shifting toward a more mature, fermentation-driven system where plant fiber degradation played a central role^49^. While this indicates proper microbial colonization, it could also imply that methanogenesis during this stage may lead to a reduced energy efficiency^50^. As elephants were transitioning from milk-based to more fiber-rich diets, the increasing methanogenesis might reflect an adaptation to higher dietary fiber intake, but it also suggested potential energy trade-offs that could affect overall growth and development during this critical period.

By 11 to 12 months, Weighted UniFrac analysis indicated no significant difference in microbial community distance between the infants and their mothers, suggesting a convergence towards a more mature microbial composition. However, despite this increasing similarity, species richness and evenness in the infants’ microbiota continue to lag behind those of the mothers. This suggested that, while the overall community structure becomes more aligned over time, the infant gut microbiota has not yet fully reached the diversity and complexity of an adult elephant. This gradual progression highlights the ongoing maturation process of the gut microbiota during the first year of life. The mother’s gut microbiota, rich in fibrolytic bacteria such as *Lachnospiraceae*, *Spirochaetaceae*, *Planococcaceae*, and *Rikenellaceae*, as well as genera like *Treponema* and *Rikenellaceae_RC9_gut_group*, reflects maternal diet focus on fibrous plant material. These fibrolytic bacteria played crucial roles in the degradation of complex plant fibers, an essential function for the adult elephant’s herbivorous diet, which the infant is gradually adopting.

## Conclusion

In conclusion, our study describes the gradual establishment and maturation of the gut microbiota in elephant calves during their first year of life. Maternal milk appears to contribute to the initial microbial colonization during the first month. Coprophagy may further increase microbial diversity by facilitating vertical transfer from mothers, potentially supporting gut microbiome stabilization and nutrient absorption between 2 and 6 months. Although calf gut microbiota composition becomes more similar to that of adults by 11 to 12 months, species richness and diversity remain lower, suggesting ongoing microbial development as infants transition from milk to more fibrous diets. The presence of bacterial and archaeal taxa associated with fiber degradation and methanogenesis indicates an evolving gut microbiota that likely plays a role in digestive process. This study focused solely on microbiome analysis without direct measures of health or development. Future research including physiological and growth parameters would help clarify the relationships between gut microbiota and host development.

## Methods

### Animals and sample collection

The experimental protocol was approved by the Institutional Animal Care and Use Committee of the Faculty of Veterinary Medicine, Chiang Mai University, Chiang Mai, Thailand (Approval No. FVM-ACUC; R3/2563). All experimental procedures were performed in accordance with relevant guidelines and regulations. This study is reported in accordance with the ARRIVE guidelines.

Fecal samples were collected from five elephant calves and their respective mothers housed in tourist elephant camps located in Chiang Mai, Thailand. All calves were born between 2019 and 2020 and were kept with their mothers in large soil and cement corrals. To minimize external variables, the elephants had no tourist interactions and were not relocated during the study. Only clinically healthy calves were included. Calves with any history of illness, medication, translocation, milk replacer feeding, or dietary supplementation were excluded. The mothers were fed a roughage-based diet consisting of maize stalks (*Zea mays L*.), Napier grass (*Pennisetum purpureum*), and seasonal local fruits such as bananas, pineapples, mangoes, and tamarinds, along with commercial elephant pellets (Erawan^®^, Charoen Pokphand Foods, Thailand).

### Fecal samples collection and bacterial DNA preparation

Because gut microbiota in elephant calves change rapidly during the transition from milk to solid plant-based diets^10^, we collected samples at one-month intervals to capture detailed temporal patterns throughout the first year of life. Fresh fecal samples were collected from each calf starting at meconium (within 1 h of birth) and then monthly for 12 months (November 2019–October 2021). Maternal fecal samples were collected when calves reached 3 months of age. Meconium samples were obtained from two calves (Ele-4 and Ele-5), while maternal samples were available for all except Ele-4 (Table 1).

**Table 1:** General characteristics and sample details of captive Asian elephant calves.

	Sex	Elephant camp	Date of Calf Birth	Sample collection end date	Meconium	Maternal samples
**Ele-1**	Female	Camp 1	Nov, 2020	Oct, 2021	-	✓
**Ele-2**	Male	Camp 1	Nov, 2020	Oct, 2021	-	✓
**Ele-3**	Male	Camp 2	Nov, 2019	Oct, 2020	-	✓
**Ele-4**	Male	Camp 2	Mar, 2020	Feb, 2021	✓	-
**Ele-5**	Female	Camp 2	Mar, 2020	Feb, 2021	✓	✓

Each fresh fecal sample and meconium, approximately 50 grams, was collected immediately after defecation from the ground using sterile disposable spatulas and placed into sterile collection containers. To minimize potential surface contamination, meconium and fecal samples were processed using a sterile sub-surface (core) sampling approach, in which the inner portion of the bolus was aseptically collected for DNA extraction. A subsample was immersed in RNAlater (Life Technologies, USA) to stabilize nucleic acids and stored at −20°C until processing. Before DNA extraction, samples were thawed and indigestible materials (grass, seeds, fruit peels) were removed. Then 250 mg of homogenized sample was subjected to DNA extraction using the QIAamp PowerFecal Pro DNA Kit (QIAGEN, Germany). The hypervariable V3-V4 region of the 16S rRNA gene was amplified using specific primers (F 5’-CCTAYGGGRBGCASCAG-3’, R 5’-GGACTACNNGGGTATCTAAT-3’). Sequencing was subsequently performed using paired-end 2 × 250 bp reads on an Illumina NovaSeq 6000 sequencing system. Raw paired-end reads were subjected to quality control, including removal of adapter and barcode sequences, paired-end read merging using FLASH. Quality filtering was performed using fastp to eliminate low-quality reads and reads containing ambiguous bases. Chimeric sequences were removed during the clean data generation step prior to downstream analysis. Sequencing quality was assessed based on Phred quality scores (Q20/Q30), GC content, and read length distribution. DNA amplification, quality control, and sequencing were conducted by Novogene Inc. (Singapore City, Singapore). A double-blind study design, along with systematic sample categorization, was employed to minimize potential biases.

### Bioinformatics and data analysis

DNA sequence data from NGS were processed using the Quantitative Insights Into Microbial Ecology 2 (QIIME 2 version 2023.2.0) open-source software^51^. Paired-end reads were denoised, merged, and trimmed using q2-dada2 plugin version 2023.2.0^52^ to generate amplicon sequence variants (ASVs). After denoising and quality filtering, sequencing depth ranged from 15,797 to 115,689 reads per sample, with a mean depth of 32,627 reads per sample. To retain all samples, diversity analyses were conducted with rarefaction at a sequencing depth of 14,500 reads per sample. A feature table including the number of each ASV per sample was also generated^53^. The ASVs were aligned with MAFFT and used to generate a phylogenetic tree for further analysis^54,55^. The diversity analyses were conducted with rarefication at the sequencing depth of 14,500 as this is the minimum reads to retain all the samples in this study^56^.

### Statistical analysis

Distance-based similarity analysis was conducted to explore the dissimilarities in microbial communities across different age groups. For alpha diversity, we calculated Chao1^57^ and Shannon’s index^58^ using the vegan package in R (v2024.04.2). To account for repeated measures from the same individual elephants, we applied Linear Mixed-Effects Models (LMMs), with Month as a fixed effect and Elephant ID as a random effect. Pairwise comparisons among age groups were conducted using the Benjamini-Hochberg (BH) method to adjust for multiple testing. In addition, Generalized Additive Mixed Models (GAMMs) were used to assess non-linear trajectories of alpha diversity over age, capturing gradual trends in community richness and evenness.

For beta diversity, Bray-Curtis, Jaccard, unweighted UniFrac^59^, and weighted UniFrac^60^ distance matrices were computed using the vegan package and visualized via Principal Coordinate Analysis (PCoA). Differences between age groups were evaluated using LMMs on PCoA axes scores, with post-hoc comparisons adjusted using the BH method. This approach allowed us to identify both linear and non-linear age-related changes in microbial diversity while accounting for repeated measures within individuals.

Differential abundance among months was assessed using LEfSe^61^, with an alpha cutoff of 0.05 and an LDA threshold > 4. Functional predictions were generated using Functional Annotation of Prokaryotic Taxa (FAPROTEX) to infer metabolic potentials from 16 S rRNA-based taxonomic profiles^62,63^.

## Supplementary Information

Below is the link to the electronic supplementary material.

Supplementary Material 1

Supplementary Material 2

Supplementary Material 3

Supplementary Material 4

Supplementary Material 5

Supplementary Material 6

Supplementary Material 7

Supplementary Material 8

## Data Availability

The raw 16 S rRNA gene sequence data and metadata files in this study are available in the NIH Sequence Read Archive under the accession number PRJNA1332645 (https:/www.ncbi.nlm.nih.gov/bioproject/PRJNA1332645).
